# Can a Conversation Between Mesenchymal Stromal Cells and Macrophages Solve the Crisis in the Inflamed Intestine?

**DOI:** 10.3389/fphar.2018.00179

**Published:** 2018-03-06

**Authors:** Laura Hidalgo-Garcia, Julio Galvez, M. Elena Rodriguez-Cabezas, Per O. Anderson

**Affiliations:** ^1^Center for Biomedical Research (CIBM), CIBER-EHD, ibs.Granada, Department of Pharmacology, University of Granada, Granada, Spain; ^2^Stromal Cells and Immunology Group, Pfizer, University of Granada, Andalusian Regional Government Centre of Genomics and Oncological Research (GENYO), Granada, Spain

**Keywords:** multipotent mesenchymal stromal cells, mesenchymal stem cells, inflammatory bowel disease, M1/M2 macrophage polarization, IL-10, PGE2

## Abstract

Inflammatory bowel disease (IBD) is a group of chronic inflammatory conditions of the gastrointestinal tract characterized by an exacerbated mucosal immune response. Macrophages play pivotal roles in the maintenance of gut homeostasis but they are also implicated in the pathogenesis of IBD. They are highly plastic cells and their activation state depends on the local environment. In the healthy intestine, resident macrophages display an M2 phenotype characterized by inflammatory energy, while inflammatory M1 macrophages dominate in the inflamed intestinal mucosa. In this regard, modifying the balance of macrophage populations into an M2 phenotype has emerged as a new therapeutic approach in IBD. Multipotent mesenchymal stromal cells (MSCs) have been proposed as a promising cell-therapy for the treatment of IBD, considering their immunomodulatory and tissue regenerative potential. Numerous preclinical studies have shown that MSCs can induce immunomodulatory macrophages and have demonstrated that their therapeutic efficacy in experimental colitis is mediated by macrophages with an M2-like phenotype. However, some issues have not been clarified yet, including the importance of MSC homing to the inflamed colon and/or lymphoid organs, their optimal route of administration or whether they are effective as living or dead cells. In contrast, the mechanisms behind the effect of MSCs in human IBD are not known and more data are needed regarding the effect of MSCs on macrophage polarization that would support the observation reported in the experimental models. Nevertheless, MSCs have emerged as a novel method to treat IBD that has already been proven safe and with clinical benefits that could be administered in combination with the currently used pharmacological treatments.

## Functional plasticity of macrophages

Macrophages are tissue resident phagocytic cells that play fundamental roles in steady-state tissue homeostasis, regulation of the inflammatory response and host defense. Macrophages respond promptly to environmental stimuli using multiple receptors that results in a specific and optimized activation state ready to deal with the task at hand (Murray, 2017).

**Figure 1:**
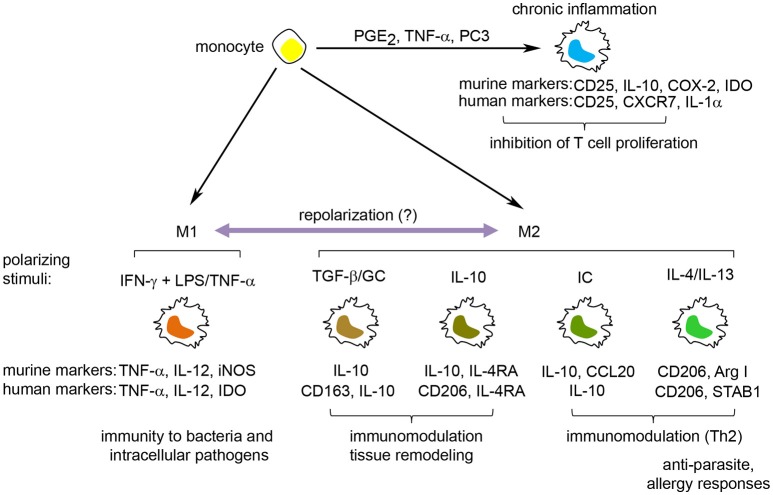
The spectrum of macrophage activation. Macrophages can respond to a wide range of stimuli, resulting in the induction of a spectrum of macrophage activation states. These include M1 macrophages, involved in the protection against bacteria, and M2 macrophages, induced by Th2 cytokines, anti-inflammatory cytokines (IL-10, TGF-β), immune complexes and glucocorticoids, and participate in anti-parasite immune responses, tissue remodeling/wound healing and inhibition of immune responses. Furthermore, stimuli associated with chronic inflammation, including PGE_2_, TNF-α and the TLR2-ligand PC3, induce a macrophage activation state distinct from the M1/M2 macrophages that have the potential to inhibit T cell proliferation. Defining molecules for murine and human M1 and M2 macrophages are indicated under each specific polarization state. GC, glucocorticoids; IC, immune complexes; IDO, indoleamine 2,3-dioxygenase; iNOS, inducible nitric oxide synthase.

The activation states of macrophages were initially divided into classically activated M1 macrophages [induced by interferon (IFN)-γ], which participate in the anti-microbial response, and alternatively activated M2 macrophages [induced by interleukin (IL)-4], which protect against parasites and participate in wound healing/tissue remodeling (Stein et al., 1992; Hill Charles et al., 2000). While the prototypic M1/M2 polarization states are clearly established *in vitro*, their distinction *in vivo* has been difficult due to the multitude of stimuli resulting in mixed M1/M2 macrophage activation states (Martinez and Gordon, 2014). Recent data points to a continuum of activation states where stimulation of macrophages with lipopolysaccharide (LPS), tumor necrosis factor (TNF)-α, IL-10, IL-13, transforming growth factor (TGF)-β, glucocorticoids (GC), or immune complexes (IC) gives rise to similar but distinct transcriptional and functional macrophage activation states along the M1-M2 axis (Martinez and Gordon, 2014; Murray et al., 2014; Xue et al., 2014; Murray, 2017). In addition, stimulation of macrophages with free fatty acids, high-density lipoprotein (HDL) or with stimuli involved in chronic inflammation [including prostaglandin (PG) E_2_ and the toll like receptor (TLR) 2 ligand P3C] results in macrophage activation states that go outside the M1-M2 continuum (Popov et al., 2008; Xue et al., 2014) showing the complexity of macrophage activation and function (Figure 1).

A large number of surface molecules, cytokines, intracellular enzymes, and transcription factors are used to identify and differentiate between discrete macrophage activation states. M1 macrophages are generally distinguished by their high production of proinflammatory cytokines (IL-6, IL-12, TNF-α) and the expression of inducible nitric oxide synthase (iNOS) (in mouse) and indolamine 2,3,-dioxygenase (IDO) (in human). Markers for M2 macrophages encompass both stimuli-specific molecules (Xue et al., 2014) and more general M2 markers, such as CD206 (mannose receptor) and arginase I (Murray et al., 2014). CD206 is a surface marker for murine (Stein et al., 1992) and human (Murray et al., 2014) M2 macrophages induced by IL-4/IL-13 or IL-10 (Mantovani et al., 2004). In contrast, arginase I expression and activity are frequently used as a marker for murine, but not human, M2-polarized macrophages (Thomas and Mattila, 2014). Finally, IL-10 is one of the most used markers for M2 macrophages due to its higher expression in several M2 macrophage polarization states (except for IL-4/IL-13-induced M2 macrophages) compared to M1 macrophages.

As mentioned above, macrophages are functionally plastic cells whose activation states are dictated by the relative concentration of M1/M2 polarizing stimuli in the local environment (Wynn et al., 2013; Smith et al., 2016). As a consequence, switches between macrophage polarization states (M1 to M2 and vice versa) can be seen during responses to infection, wound healing and disease, including cancer (Qian and Pollard, 2010; Wynn et al., 2013). However, it is not clear whether these changes in macrophage activation status are due to (i) recruitment of new monocytes and their subsequent activation in response to changed local cues or (ii) repolarization of M1 macrophages into M2 macrophages or vice versa, or (iii) a combination of both (Italiani and Boraschi, 2014). While the repolarization of M1 into M2 macrophages has been described (Porcheray et al., 2005; Davis et al., 2013; Tarique et al., 2015; Kudlik et al., 2016), a recent study showed that human and murine M1 macrophages failed to convert into M2 cells upon IL-4 exposure *in vitro* and *in vivo* due to mitochondrial dysfunction (Van Den Bossche et al., 2016).

## Role of macrophages in IBD

Inflammatory bowel disease (IBD) is a group of chronic gastrointestinal inflammatory diseases that include Crohn's disease (CD) and ulcerative colitis (UC). They both feature alternating periods of remissions and relapses, characterized by uncontrolled intestinal inflammation, with disabling symptoms like diarrhea, abdominal pain, fever, clinical signs of bowel obstruction, as well as passage of blood or mucus or both. This implies extended medical and/or surgical procedures that impair the patients' quality of life. At present, the etiology of IBD is not fully elucidated, and most probably results from an intricate combination of four major factors: genetic predisposition, compositional and metabolic changes in the intestinal microbiota (dysbiosis), environmental exposures, and deregulation of mucosal immune responses (de Souza et al., 2017).

There is a consensus that IBD appears in genetically susceptible individuals who display an altered intestinal barrier function with increased paracellular permeability. These patients develop an exaggerated immune response toward the intestinal microbiota that triggers the chronic intestinal inflammation. In this scenario, cells from both the innate [including intestinal epithelial cells, monocytes/macrophages, neutrophils, and dendritic cells (DCs)] and adaptive (including T- and B-cells) arms of the mucosal immune system and their secreted mediators (cytokines, chemokines, eicosanoids and reactive oxygen and nitrogen species) are involved in the pathogenesis of IBD (Xavier and Podolsky, 2007).

The largest population of macrophages in the body resides in the gastrointestinal mucosa (Lee et al., 1985), where they play pivotal roles in the maintenance of epithelial and immunological homeostasis (Pull et al., 2005; Isidro and Appleyard, 2016). The intestinal macrophage pool is continuously replenished from Ly6C^high^ monocyte precursors recruited in a CCR2-dependent manner into the intestinal lamina propria (Bain et al., 2014). In steady state, the local microenvironment of the intestinal mucosa induces monocytic precursors to acquire the homeostatic phenotypic properties of intestinal-resident macrophages (M2 phenotype). These intestinal macrophages display scavenger and bacteriocidal activities together with an inflammatory anergy which is, in part, induced by commensal microbiota (Ueda et al., 2010; Zigmond et al., 2014) and intestinal stromal cell cues (Smythies et al., 2005; Maheshwari et al., 2011). They are also characterized by an anti-inflammatory gene expression profile that involves the up-regulation of IL-10, a cytokine with anti-inflammatory properties (Bain and Mowat, 2014). Importantly, IL-10 plays a key role in regulating the pro-inflammatory responses of murine and human intestinal macrophages and mutations in its receptor, IL-10R, result in acute IBD in humans (Glocker et al., 2009) and severe spontaneous colitis in mice (Zigmond et al., 2014). However, in human IBD and murine experimental colitis, the CD14^+^ monocytes that are recruited into the inflamed colonic mucosa fail to become anergic. Instead, they turn into inflammatory macrophages (M1 phenotype) that produce high levels of proinflammatory cytokines (including IL-1β, TNF-α, IL-23), nitric oxide and reactive oxygen intermediates (Grimm et al., 1995; Tokuyama et al., 2005; Joeris et al., 2017). Proinflammatory CD14^+^ macrophages also home to the mesenteric lymph nodes (MLN) where they promote disease (Li et al., 2017). In this regard, the inflammatory macrophages outnumber the resident population, and all the secreted pro-inflammatory mediators have a deleterious impact on epithelial permeability, increasing pathogen invasion (Du Plessis et al., 2013) and promoting accumulation of IL-17-producing innate and adaptive leukocytes (Coccia et al., 2012).

## Modulation of macrophage activation as a treatment for IBD

Considering all the above, the pharmacological alteration of the balance of macrophage populations in the inflamed intestine, especially promoting an increase in the anti-inflammatory M2 phenotype, is becoming an attractive therapeutic approach in IBD. Firstly, administration of *in vitro* generated M2 macrophages, secreting high levels of IL-10, has been found to lessen the severity of colitis in mice (Hunter et al., 2010; Anderson et al., 2013b; Leung et al., 2013). Secondly, mice infected with schistosome worms acquired a macrophage-dependent protection against DSS-induced colitis. This protection was not associated with any known M2 macrophage activation state or IL-10/TGF-β expression. However, transfer of colon lamina propria macrophages from infected mice significantly suppressed colitis in recipient mice (Smith et al., 2007), highlighting the multitude of immunomodulatory macrophage activation states. Thirdly, patients with active CD showed fewer CD68^+^CD206^+^ macrophages in the inflamed mucosa (which indicates alternatively activated macrophages) than patients with inactive CD (Hunter et al., 2010). Interestingly, the anti-TNF-α monoclonal antibody, infliximab, which is successfully used in the treatment of human IBD (Danese et al., 2015), was found to induce regulatory macrophages (CD68^+^CD206^+^) *in vitro* (Vos et al., 2011) and in patients with IBD responding to treatment (Vos et al., 2012).

## Multipotent mesenchymal stromal cells

**Figure 2:**
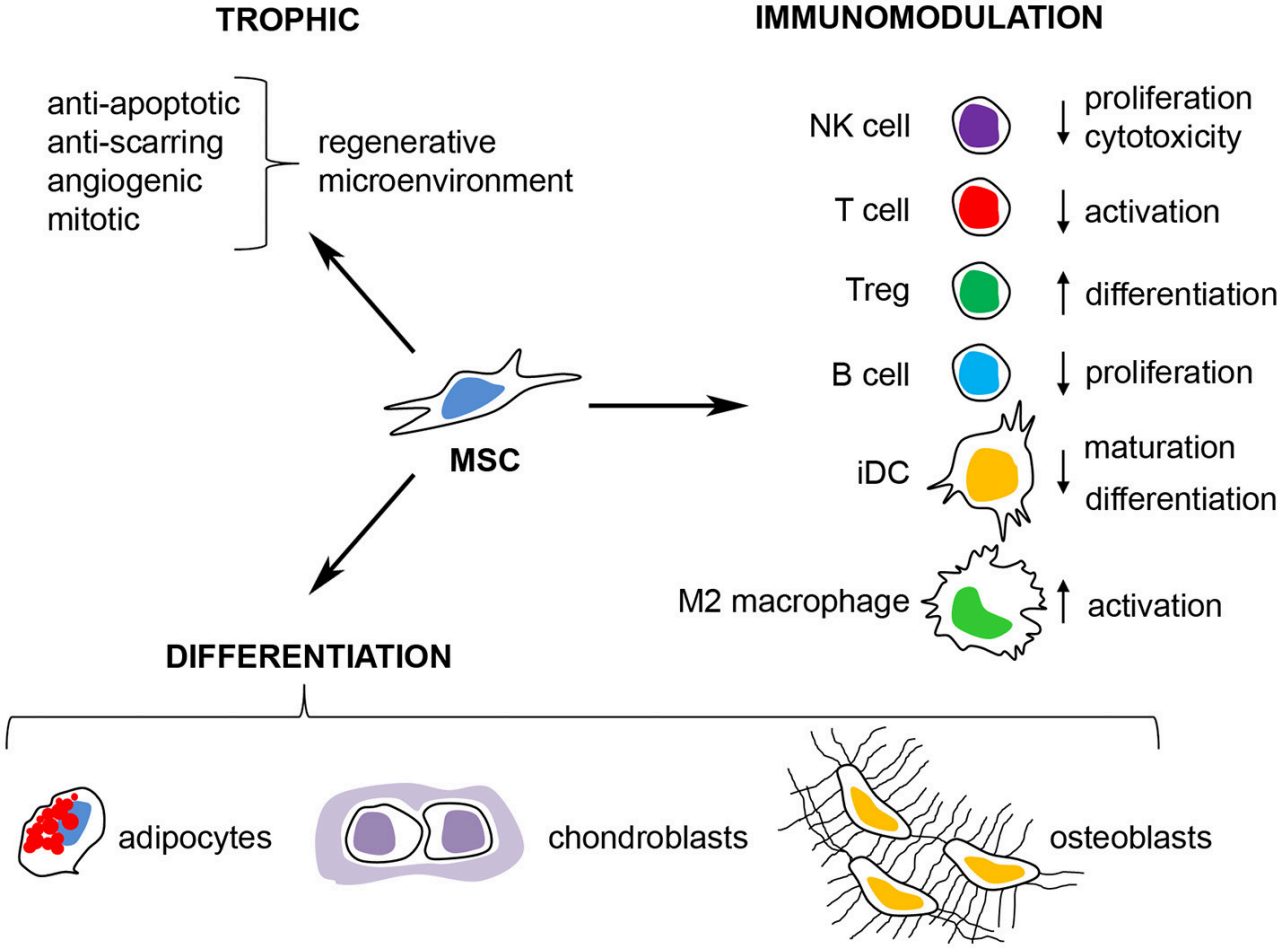
Therapeutic properties of multipotent mesenchymal stromal cells. MSCs have emerged as a promising cell therapy for inflammatory/autoimmune diseases and in regenerative medicine due to their (i) secretion of trophic factors that promote a regenerative microenvironment, (ii) their capacity to differentiate into adipocytes, osteoblasts, and chondroblasts *in vitro* and *in vivo* and (iii) their immunomodulatory capacity where MSCs can inhibit the activation of T cells, NK cells, and B cells, prevent the maturation of dendritic cells (iDC) and promote immunological tolerance through the induction of M2 macrophages and regulatory T cells (Tregs).

Multipotent mesenchymal stromal cells (MSCs) are non-hematopoietic, perivascular cells with tissue regenerative and immunomodulatory abilities that have emerged as a promising cell-therapy for regenerative medicine, autoimmune disease and cancer. The interest in MSCs began when Friedenstein originally described the existence of a rare non-hematopoietic bona fide stem cell in the bone marrow that could give rise to multiple skeletal tissues (bone, cartilage, and fibrous tissue) when transplanted *in vivo* (reviewed in Friedenstein, 1990). These cells were designated osteogenic stem cells or bone marrow (BM) stromal stem cells and were later found to promote bone remodeling and hematopoiesis *in vivo* (Bianco et al., 2008; Méndez-Ferrer et al., 2010). Subsequently, cells with similar morphology and *in vitro* differentiation potential were isolated by plastic adherence from several adult and neonatal tissues and organs, including adipose tissue, muscle, umbilical cord and placenta (da Silva Meirelles, 2006). These cells were named “multipotent mesenchymal stromal cells,” to distinguish them from the osteogenic stem cells/BM stromal stem cells described by Friedenstein (Owen and Friedenstein, 1988). The current minimal criteria to define human MSCs are (i) plastic adherence under normal culture conditions *in vitro* (ii) expression of CD73, CD90 and CD105 and lack of expression of CD45, CD34, CD14 or CD11b, CD79α or CD19 and HLA-DR surface molecules and (iii) differentiation into osteoblasts, adipocytes and chondroblasts *in vitro* (Dominici et al., 2006). Although the *in vivo* function and differentiation potential of MSCs appear to depend on their tissue of origin (Sacchetti et al., 2016), *in vitro* expanded MSCs can migrate to sites of injury/inflammation (Kidd et al., 2009), secrete trophic factors and are potent regulators of the innate and adaptive immune responses *in vitro* and *in vivo* (Di Nicola et al., 2002; Constantin et al., 2009; Gonzalez-Rey et al., 2009; Anderson et al., 2013a, 2017; Gao et al., 2016) (Figure 2). Several clinical trials have evaluated the immunomodulatory and tissue regenerative potential of both autologous and allogeneic MSCs with promising results (Quarto et al., 2001; Connick et al., 2012; von Bahr et al., 2012).

## MSCs and their potential application as a cell therapy for IBD

MSCs are nowadays considered a promising future treatment of IBD. Administration of syngeneic, allogeneic and xenogeneic MSCs ameliorates experimental colitis by regulating Th1/Th17 responses, inducing regulatory T cells (Tregs) and reducing the levels of proinflammatory cytokines in the inflamed colon (Hayashi et al., 2008; Gonzalez-Rey et al., 2009; Li et al., 2013). Clinical trials using MSCs for the treatment of fistulizing CD (Ciccocioppo et al., 2011; Panés et al., 2016; Dietz et al., 2017), luminal CD (Duijvestein et al., 2010; Forbes et al., 2014; Zhang et al., 2017) and UC (Hu et al., 2016) have shown encouraging clinical responses and safety.

### Effect of MSCs on macrophage polarization *in vitro*

Numerous studies have demonstrated that MSCs can induce immunomodulatory M2-like macrophages *in vitro* that can inhibit T cell and NK cell function and induce Tregs (Gonzalez-Rey et al., 2009; Anderson et al., 2013b; Melief et al., 2013a; Chiossone et al., 2016). Conditioned medium of murine adipose tissue-derived MSCs (ASCs) was found to induce regulatory macrophages *in vitro*, which were distinct from IL-4 activated macrophages. These ASC-induced macrophages were characterized by high arginase I activity, IL-10 production and expression of LIGHT, heme oxygenase (HO)-1 and arginase II, possessing immunomodulatory capacity *in vitro* and in experimental colitis and sepsis (Anderson et al., 2013b). Furthermore, murine BM-MSCs have been shown to induce high IL-10 production in both M-CSF-derived BM-macrophages and in thioglycollate-induced peritoneal macrophages (Cho et al., 2014; Kudlik et al., 2016). Importantly, some studies have suggested that MSCs can repolarize M1 macrophages (induced by M-CSF + LPS or GM-CSF + IFN-γ) into IL-10 expressing M2 macrophages (Németh et al., 2009; Manferdini et al., 2017). In general, the main MSC-derived molecule that promotes the M2 activation state is PGE_2_, although other effector molecules could also be involved (Table 1).

**Table 1:** Effect of different MSC mediators on macrophage polarization *in vitro* and *in vivo*.

**MSC SOURCE MODULATION** ***IN VITRO***					
**MSC source**	**Macrophage source**	**Induction**	**Implicated mediator**	**Macrophage activation state**	**References**
Murine BM-MSCs	Murine BM	M-CSF	ND	IL-10^high^iNOS activity^down^/Arg activity^up^	Cho et al., 2014
Murine BM-MSCs	Murine BM	M-CSF	PGE_2_	IL-10^high^TNF-α^low^	Kudlik et al., 2016
Murine BM-MSCs	Peritoneal macrophages	Thioglycollate	PGE_2_	IL-10^high^IL-12p40^high^IL-6^low^TNF-α^low^	Maggini et al., 2010
Murine ASCs and conditioned media	Murine BM	M-CSF	PGE_2_	IL-10^high^Arg activity^up^LIGHT^+^	Anderson et al., 2013b
Human BM-MSCs	Human CD14^+^ blood monocytes		IDO	IL-10^high^CD206^+^	François et al., 2012
Human BM-MSCs	PBMCs	M-CSF	PGE_2_	IL-10^int^/TNF-α^low^/IL-6^low/^TGF-β^high^/DC-SIGN^int^/CD206^int^	Vasandan et al., 2016
Human BM-MSCs	Human CD14^+^ blood monocytes		ND	IL-10^high^CCL18^+^ CD206^+^CD163^+^	Melief et al., 2013a
Human BM-MSCs	Human CD14^+^ blood monocytes	M-CSF	ND	IL-10^high^CD206^+^	Chiossone et al., 2016
Human amniotic MSCs (and their CM)	Human CD14^+^ blood monocytes	GM-CSF	PGE_2_	IL-10^high^CD206^+^DC-SIGN^+^	Magatti et al., 2017
Murine ASC-derived exosomes	Murine BM	L929 CM (30%)	ND	IL-10^high^CD206^+^IL-6^low^iNOS activity^down^/ArgI activity^up^	Henao Agudelo et al., 2017
Human ASC-derived exosomes	Murine BM	GM-CSF	ND	CD206^+^CD36^+^CD86^−^CD40^−^	Lo Sicco et al., 2017
Human UC-MSC-derived exosomes	Peritoneal macrophages		ND	IL-10^high^TNF-α^low^IL-6^low^(qPCR)IL-7^low^	Mao et al., 2017a
Human UC-MSC-derived exosomes	Murine BM	M-CSF	miR-146a	IL-10^high^ArgI^+^TNF-α^low^iNOS^low^	Song Y. et al., 2017
**MACROPHAGE MODULATION** ***IN VIVO***					
**MSCs source**	**Animal model**	**Administration**	**Implicated mediator**	**Macrophage activation state**	**References**
Murine BM-MSCs	DSS colitis	i.p. 3 × 10^6^ cells on day 5	TSG-6	ArgII^+^CCL22^+^HO-1^+^TNF-α^low^ IL-12^low^	Sala et al., 2015
Murine BM-MSCs	DSS colitis	i.v. 1 × 10^6^ cells on day 8	ND	F4/80^+^CD206^+^	Wang et al., 2014
Murine ASCs	DSS colitis	i.p. 1 × 10^6^ cells on day 1 and 5	ND	ArgI increased in colon (protein and mRNA)	de Aguiar et al., 2018
Murine BM- MSCs	DSS colitis	i.p. 0.5 × 10^6^ cells on day 0 and 12	Knock down of gal-3	IL-10^high^IL-12^low^ F4/80^+^CD206^+^	Markovic et al., 2015
Murine BM-MSCs	TNBS colitis	i.v. 1 × 10^6^ cells on day 0	ND	CD11b^+^splenic macrophages	Parekkadan et al., 2011
Human ASCs	DSS colitis	i.p. 2 × 10^6^ cells on day 1	TSG-6	CD206^+^ArgI^+^/ym1^+^/Fizz1^+^	Song W. J. et al., 2017
Human UC-MSC extracts	DSS colitis	i.p. 150μg/mouse on day 3	ND	ArgI^+^LIGHT^+^CCL1^+^ in peritoneal macrophages	Song J. et al., 2017
**INHIBITION OF MONOCYTE TO iDC DIFFERENTIATION**					
**MSC source**	**Macrophage source**	**iDC induction**	**Implicated mediator**	**Macrophage activation state**	**References**
Human BM-MSCs	Human CD14^+^ blood monocytes	IL-4+ GM-CSF	IL-6	CD14^+^CD1a^−^IL-10^high^	Melief et al., 2013a
Human UC-MSCs	Human CD14^+^ blood monocytes	IL-4+ GM-CSF	Lactate	CD14^+^CD1a^−^IL-10^high^TGF-β1^+^IL-6^+^	Selleri et al., 2016
Human UC-MSC	Human CD14^+^ blood monocytes	IL-4+ GM-CSF	IL-6, HGF	CD14^+^CD1a^−^IL-10^high^IL-12^low^	Deng et al., 2016

*ND, not determined; Gal-3, galectin-3; Arg, arginase; i.p., intraperitoneal; i.v., intravenous; PBMCs, peripheral blood mononuclear cells; BM, bone marrow; UC, umbilical cord; CM, conditioned medium; CD163, hemoglobin scavenger receptor; LIGHT, TNF superfamily member 14; DC-SIGN, CD209/dendritic cell-specific intercellular adhesion molecule-3-grabbing non-integrin; CCL1, C-C motif chemokine ligand 1; CCL18, C-C motif chemokine ligand 18; CCL22, C-C motif chemokine ligand 22; HO-1, heme oxygenase-1; ym1, chitinase-3-like protein 3; Fizz1, found in inflammatory zone 1; IDO, indolamine 2,3,-dioxygenase*.

Similarly, human ASCs (Manferdini et al., 2017), BM-MSCs (Melief et al., 2013a; Chiossone et al., 2016; Vasandan et al., 2016), and amniotic MSCs (Magatti et al., 2017) induced an M2 activation state in CD14^+^ monocytes (stimulated or not with either M-CSF or GM-CSF). In all cases, the M2 activation state was characterized by CD206 expression and IL-10 secretion and when analyzed, depended on MSC-derived PGE_2_ (Table 1). One study also implicated IDO activity in MSCs in the induction of CD206^+^IL-10^high^ M2 macrophages (François et al., 2012). However, IDO is also expressed in some types of M2-like macrophages that could make the effects of pharmacological inhibition of IDO on MSC-mediated macrophage polarization difficult to interpret (Xue et al., 2014; Selleri et al., 2016).

In addition, exosomes from murine and human MSCs have been shown to induce IL-10^high^CD206^+^ macrophages (Henao Agudelo et al., 2017; Lo Sicco et al., 2017; Mao et al., 2017a). Song et al. found that human umbilical cord (UC)-MSC-derived exosomes transferred microRNA (miR)-146a to macrophages, inducing an M2 phenotype (Song Y. et al., 2017). Interestingly, Phinney et al. showed that MSCs exposed to oxidative stress shed exosomes containing depolarized mitochondria, which are taken up by macrophages. However, in order to prevent an TLR-mediated inflammatory response to the engulfed mitochondria, MSCs simultaneously shed miR-containing exosomes that repress TLR signaling and production of inflammatory mediators in macrophages (Phinney et al., 2015).

MSCs can also prevent the GM-CSF/IL-4-induced differentiation of monocytes into immature dendritic cells (iDCs) *in vitro*. Here, the presence of MSCs promotes a CD14^+^CD1a^−^IL-10^high^ “M2-like” macrophage activation state with low allostimulatory capacity. To date, several MSC-derived mediators have been implicated in this process, including hepatocyte growth factor (HGF) (Deng et al., 2016), IL-6 (Melief et al., 2013a), and lactate (Selleri et al., 2016).

**Figure 3:**
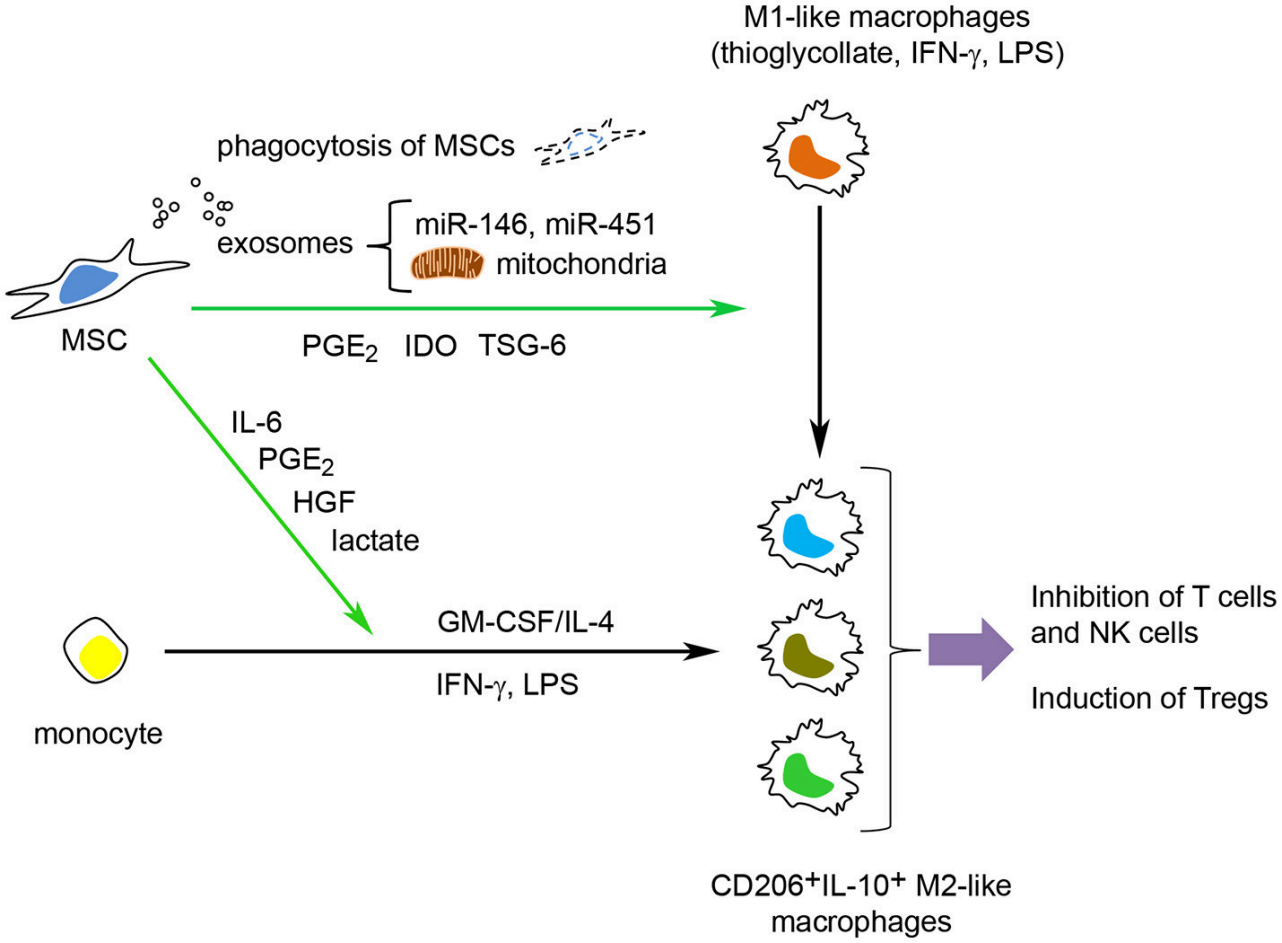
MSCs can modulate macrophage function through several mechanisms. MSCs can induce M2 polarization of monocytes, primed M1 macrophages (stimulated with GM-CSF or thioglycollate) and polarized M1 macrophages (polarized with LPS and/or IFN-γ). MSCs can also inhibit the GM-CSF/IL-4-mediated differentiation of monocytes into immature dendritic cells and prevent the LPS/IFN-γ-mediated polarization into M1 macrophages and instead promote a M2-like macrophage activation state. MSCs use both active (TSG-6, PGE_2_, cytokines/growth factors and lactate) and passive (phagocytosis of MSCs by macrophages, transfer of microRNAs and mitochondria to macrophages) mechanisms to modulate macrophage function.

In summary, MSCs can induce M2 polarization of monocytes, primed M1 macrophages (by GM-CSF or thioglycollate) and polarized M1 macrophages (by LPS and/or IFN-γ). MSCs also promote an M2-like macrophage activation state when in contact with monocytes under dendritic cell stimuli (GM-CSF/IL-4) and M1 stimuli (LPS + IFN-γ) (Figure 3).

### Effects of MSCs on macrophage polarization *in vivo*

MSCs have been revealed to modulate macrophage polarization in models of inflammatory/autoimmune diseases and tissue regeneration (Carty et al., 2017), including sepsis (Németh et al., 2009) wound healing (Zhang et al., 2010) and spinal cord injury (Nakajima et al., 2012). Several studies have linked the therapeutic efficacy of MSCs in experimental colitis to the induction/increase of macrophages with an M2-like phenotype (Liu et al., 2015; Markovic et al., 2015; Mao et al., 2017b; de Aguiar et al., 2018). Song W. J. et al. (2017), showed that human ASCs, through their secretion of tumor necrosis factor-stimulated gene (TSG)-6, induced M2 macrophage polarization in the colon and reduced disease severity in DSS-induced colitic mice. Parekkadan et al. reported that intravenous injection of BM-MSCs induced CD4^+^foxp3^+^ Tregs and prevented TNBS-induced colitis. Chemical (clodronate treatment) or surgical depletion (splenectomy) of splenic CD11b^+^ cells abolished the therapeutic effect of the MSCs. Moreover, the injection of CD11b^+^ macrophages co-cultured with MSCs, but not skin fibroblasts, also reduced colitis (Parekkadan et al., 2011).

Supporting the above, several reports have demonstrated that MSCs injections can inhibit the infiltration of macrophages into the inflamed colon. de Aguiar et al. described that the administration of murine ASCs into mice during the induction of DSS colitis reduced the infiltration of CD11b^+^F4/80^+^ macrophages into the MLNs and colonic lamina propria. Also, MSC-injection increased the protein levels of arginase-1 in the colon, suggestive of a M2 macrophage induction (de Aguiar et al., 2018). Similarly, the infiltration of monocytes/macrophages into the inflamed colon was significantly decreased in human amnion-MSC-treated rats (Onishi et al., 2015) and in human UC-MSC-treated DSS-colitic mice (Mao et al., 2017b).

## Is the homing and viability of MSCs important for their therapeutic effects in colitis/IBD?

Injected MSCs have been shown to home to both the inflamed/injured colon and/or the MLNs and spleens in colitic mice (Gonzalez-Rey et al., 2009; Liang et al., 2011; Parekkadan et al., 2011; Castelo-Branco et al., 2012; Fan et al., 2012; Mao et al., 2017b; Takeyama et al., 2017). However, some recent studies have reported that only a minor fraction (0.001–1%) of the injected MSCs actually reaches the inflamed colon. The lack of homing in different studies can be due to inadequate culture conditions (De Becker and Van Riet, 2016), prolonged trypsinization (Chamberlain et al., 2008) or high passage number of the MSCs, since they lose the expression of chemokine receptors upon *in vitro* culturing (Honczarenko et al., 2006). Activation of MSCs using inflammatory cytokines, like IFN-γ, TNF-α, and IL-1β, has been demonstrated to induce their surface expression of chemokine receptors and integrins (Duijvestein et al., 2011b; De Becker and Van Riet, 2016). Preactivation of MSCs with IL-1β upregulated the expression of CXCR4 and increased their homing to the spleen, MLNs and the inflamed colon of DSS-treated mice (Fan et al., 2012). Duijvestein et al. also showed that pretreatment with IFN-γ increased the homing of MSCs to the inflamed intestine and potentiated their therapeutic effect in both DSS- and TNBS-induced colitis (Duijvestein et al., 2011b).

However, several recent reports suggest that the migration of MSCs to the inflamed colon and/or lymphoid organs is not necessary for their therapeutic effect in experimental colitis. Instead, intraperitoneally injected MSCs can remain trapped in the peritoneal cavity, causing a suppression of the intestinal inflammation (Bazhanov et al., 2016; Song Y. et al., 2017). In this line, Sala et al. found that intraperitoneal administration of MSCs reduced DSS-induced colitis via production of TSG-6, independently of their homing to the intestine. In fact, the injected MSCs formed aggregates in the peritoneal cavity, which remained external to the bowel wall vessels, but able to promote the induction of colonic macrophages with a regulatory phenotype (CD11b^+^F4/80^+^IL-10^high^iNOS^low^) (Sala et al., 2015).

As discussed above, the therapeutic potential of MSCs in intestinal inflammation has been linked to their capacity to actively modulate the immune system, either in lymphoid organs and the inflamed colon or through immunomodulation in the peritoneal cavity. However, it is far from clear whether MSCs are effective *in vivo* as living or dead/dying cells (Bianco et al., 2013; Sacchetti et al., 2016), and some recent studies propose that MSCs can modulate macrophage function through passive mechanisms. Luk et al. showed that heat-inactivated MSCs could not inhibit T cell and B cell activation/proliferation but could modulate monocyte function in response to LPS (Luk et al., 2016). In another study, Braza et al. found that intravenously injected MSCs were phagocytosed by murine lung macrophages that acquired a IL-10^high^TGF-β^high^IL-6^low^ phenotype in comparison to macrophages that had not ingested MSCs (Zigmond et al., 2014; Braza et al., 2016). In the same line, Song et al. described that intraperitoneal injections of freeze-thaw extracts from human UC-MSCs induced M2 polarization of intraperitoneal macrophages and reduced DSS-colitis (Song J. et al., 2017). In summary, MSCs can induce M2 macrophages *in vivo* through active or passive mechanisms, regardless of their homing to the inflamed colon and secondary lymphoid organs.

In contrast to the plethora of information on how MSCs modulate the immune system in murine colitis, the mechanisms behind their effect in human IBD are not known. Due to technical difficulties, only a few clinical studies have included sample collection in order to evaluate inflammation and immune responses in IBD patients receiving either local or systemic injections of MSCs. Hu et al. showed that administration of human UC-MSCs reduced the histological score in patients with UC, as evidenced by the improvement of the mucosal surface, mucin content in goblet cells, crypt abscesses, gland collapse, and reduction of the inflammatory infiltrate (Hu et al., 2016). Also, a decreased amount of pro-inflammatory cytokines (TNF-α, IL-1β) was observed in mucosal biopsies in patients with refractory luminal CD receiving intravenous infusions of autologous BM-MSCs (Duijvestein et al., 2010). Taken together, although the beneficial effects of MSCs have been reported in human IBD, data is lacking on the effect of MSCs on macrophage polarization that support the observations reported in preclinical studies in experimental models of rodent colitis.

## Advantages and disadvantages using MSCs for IBD

Current treatments for IBD include aminosalicylates, corticosteroids, immune-suppressants, antibiotics, and biological drugs (Bernstein, 2015). Despite their efficacy in patients with either UC or CD, their chronic use is frequently associated with severe side effects, including osteoporosis/osteonecrosis, infectious complications and development of lymphoma among others (Rutgeerts et al., 2005; Siegel, 2011; Garg et al., 2014). For this reason, there is a real need for the development of new treatments combining efficacy and safety, which could be the case of MSCs.

The main advantages of using MSCs for the treatment of IBD are: (i) administration of MSCs is safe with clinical benefits for the treatment of IBD, especially fistulizing CD. (ii) MSCs are easily isolated and can be efficiently cultured *in vitro*. (iii) MSC function is not altered by the pharmacological treatments used in IBD (azathioprine, methotrexate, 6-mercaptopurine and biologicals like anti-TNF-α), thus supporting their use in combination with these drugs (Duijvestein et al., 2010, 2011a). (iv) MSCs could be an option for CD patients refractory to current drug treatments. Interestingly, it has been shown that MSCs-treated patients can also regain responsiveness to those drugs (Ciccocioppo et al., 2015). (v) MSCs can migrate to the inflamed colon and lymphoid organs. Some studies have reported low homing efficiency probably due to the entrapment of MSCs in lungs and loss of homing receptors during *in vitro* expansion. Pretreatment of MSCs with inflammatory cytokines improves their homing to the inflamed intestine and enhances their therapeutic efficacy in experimental colitis. However, migration to the colon might not be necessary for their therapeutic effect.

Nevertheless, there are some aspects that need to be solved in order to definitively establish the therapeutic use of MSCs: (i) It is not clear whether autologous MSCs from patients with IBD possess the same therapeutic capacity as MSCs from healthy donors (Chinnadurai et al., 2015; Serena et al., 2017). A solution could be the use of allogeneic MSCs, which are considered immune evasive due to their low or absent expression of MHC class I and II and their immunosuppressive nature (Ankrum et al., 2014; Molendijk et al., 2015; Panés et al., 2016). However, IFN-γ can increase the expression of both MHC class I and II on MSCs (Romieu-Mourez et al., 2007) that would make allogeneic MSCs more susceptible to rejection in an immune-competent host. This could be a problem for applications where long term engraftment of MSCs is necessary for a clinical effect. (ii) Although MSCs administration can increase the pool of Tregs, some long term follow up studies (>6 months after intervention) suggest that the beneficial effects of the MSC-therapy wear off with time (Dave et al., 2017). This indicates a failure of establishing immunological tolerance and suggests the necessity of repeated treatments. (iii) No transformation of MSCs has been detected in any of the patients that have received MSCs injections in clinical trials. However, due to the reported pro-tumorigenic effect of MSCs *in vitro* and *in vivo* (Wei et al., 2015; Chen et al., 2017), further studies need to address whether infusion of MSCs increases the rate of colorectal cancer in IBD patients that already have an increased risk (Ullman and Itzkowitz, 2011). (iv) Moreover, since MSCs promote intestinal immunotolerance, there is a risk of infection after the cell administration. This could be addressed by the use of anti-microbial drugs (Wei et al., 2017).

In summary, MSCs can be considered as an attractive therapeutic strategy for the efficient and safe management of human IBD. Although the exact mechanisms involved require further clarification, the impact of macrophage polarization toward the anti-inflammatory M2 phenotype seems to be especially relevant.

## Author contributions

All authors listed have made a substantial, direct and intellectual contribution to the work, and approved it for publication.

### Conflict of interest statement

The authors declare that the research was conducted in the absence of any commercial or financial relationships that could be construed as a potential conflict of interest.
